# The potential and promise for clinical application of adoptive T cell therapy in cancer

**DOI:** 10.1186/s12967-024-05206-7

**Published:** 2024-05-01

**Authors:** Yinqi Li, Yeteng Zheng, Taiqing Liu, Chuanyun Liao, Guobo Shen, Zhiyao He

**Affiliations:** 1 Department of Pharmacy, Cancer Center and State Key Laboratory of Biotherapy, West China Hospital, Sichuan University, No. 37 Guo Xue Xiang, Chengdu, 610041 China; 2 Department of Biotherapy, Cancer Center and State Key Laboratory of Biotherapy, West China Hospital, Sichuan University, No. 37 Guo Xue Xiang, Chengdu, 610041 China; 3 Key Laboratory of Drug-Targeting and Drug Delivery System of the Education Ministry and Sichuan Province, Sichuan Engineering Laboratory for Plant-Sourced Drug and Sichuan Research Center for Drug Precision Industrial Technology, West China School of Pharmacy, Sichuan University, Chengdu, 610041 China

**Keywords:** Immunotherapy, Adoptive cell therapy, Chimeric antigen receptor, T cell receptor, Tumor-infiltrating lymphocytes

## Abstract

Adoptive cell therapy has revolutionized cancer treatment, especially for hematologic malignancies. T cells are the most extensively utilized cells in adoptive cell therapy. Currently, tumor-infiltrating lymphocytes, T cell receptor-transgenic T cells and chimeric antigen receptor T cells are the three main adoptive T cell therapies. Tumor-infiltrating lymphocytes kill tumors by reinfusing enlarged lymphocytes that naturally target tumor-specific antigens into the patient. T cell receptor-transgenic T cells have the ability to specifically destroy tumor cells via the precise recognition of exogenous T cell receptors with major histocompatibility complex. Chimeric antigen receptor T cells transfer genes with specific antigen recognition structural domains and T cell activation signals into T cells, allowing T cells to attack tumors without the assistance of major histocompatibility complex. Many barriers have been demonstrated to affect the clinical efficacy of adoptive T cell therapy, such as tumor heterogeneity and antigen loss, hard trafficking and infiltration, immunosuppressive tumor microenvironment and T cell exhaustion. Several strategies to improve the efficacy of adoptive T cell therapy have been explored, including multispecific chimeric antigen receptor T cell therapy, combination with immune checkpoint blockade, targeting the immunosuppressive tumor microenvironment, etc. In this review, we will summarize the current status and clinical application, followed by major bottlenecks in adoptive T cell therapy. In addition, we will discuss the promising strategies to improve adoptive T cell therapy. Adoptive T cell therapy will result in even more incredible advancements in solid tumors if the aforementioned problems can be handled.

**Graphical abstract:**

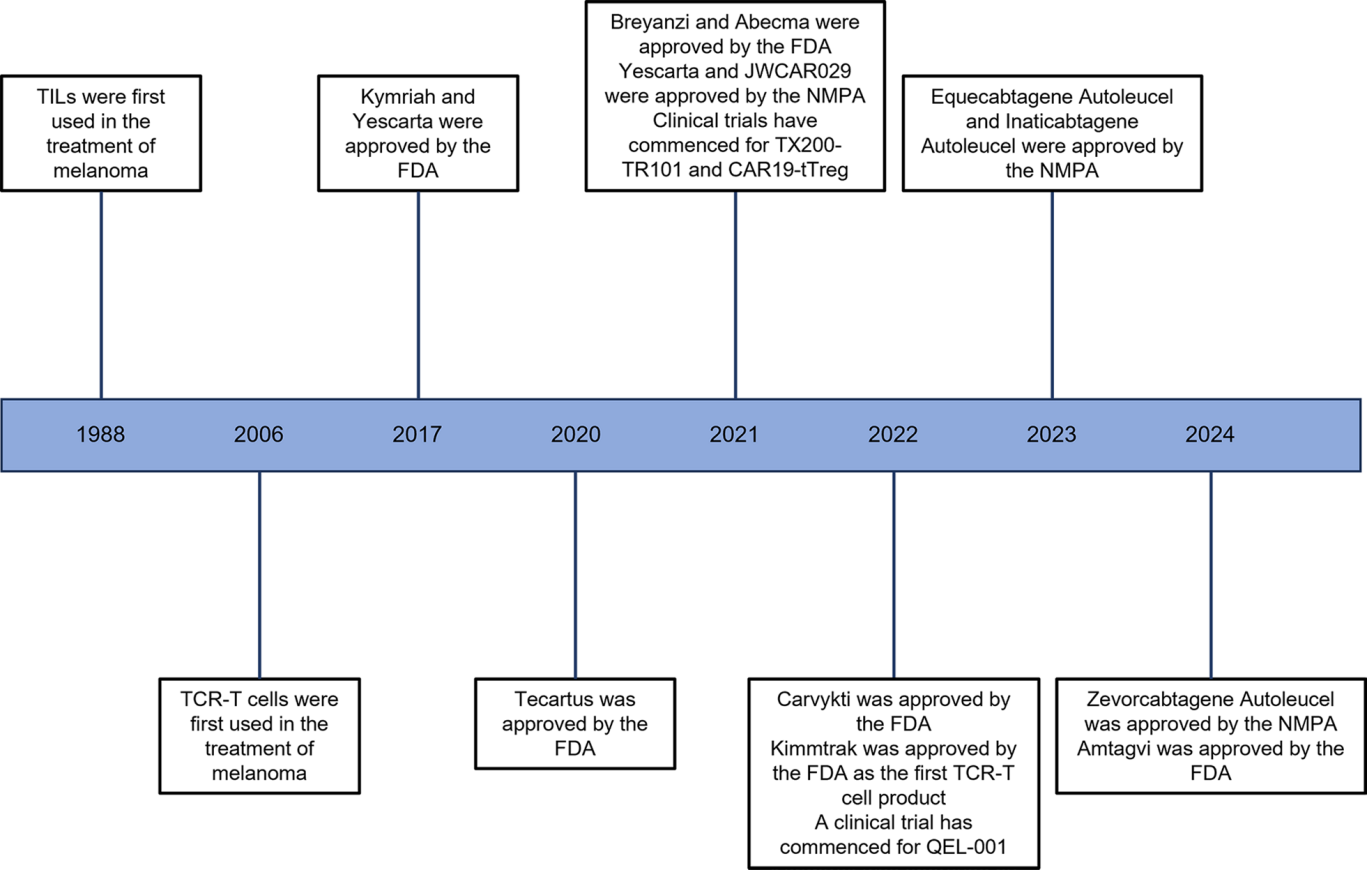

## Background

In addition to surgery, radiation and chemotherapy, immunotherapy is now playing an essential role in cancer treatment. In 2013, the world authoritative journal *Science* listed tumor immunotherapy as the most important scientific advance [1]. As a result of immunotherapy, cancer treatments have been transformed in the past ten years. Clinical trials have shown that blocking cytotoxic T lymphocyte antigen 4 (CTLA-4) and programmed cell death 1 (PD-1) could improve overall survival (OS) in advanced melanoma, which were first respectively approved by the US Food and Drug Administration (FDA) in 2011 and 2014 [2, 3]. Despite immune checkpoint inhibitor (ICI), a novel cancer immunotherapy, has provided unprecedented results in cancer treatment, there is still potential for improvement [4].

Adoptive cell therapy (ACT) was found to be highly effective for cancer immunotherapy, which has aroused intense research. Autologous or allogeneic immune cells were cultured and modified in a lab setting to improve their capacity for targeted killing before being reinfused into the patient [5]. Upon trafficking to the tumor, these cells would destroy it. ACT enhanced the ability of tumor antigens to be recognized by effector cells which could kill tumor availably [6]. This review will primarily introduce T cell-based ACT.

Tumor infiltrating lymphocytes (TILs) isolated from patients with metastatic melanoma were infused into tumors to initiate this therapeutic strategy, showing promising antitumor properties [7]. The first attempt to use chimeric antigen receptor (CAR) T cells, targeting CD19 and leading to the complete remission of relapsed and refractory leukemia, was encouraging [8]. ACT has shown the best clinical results for CD19^+^ leukemias and lymphomas when CAR T cells were used [9–11]. By genetically transferring the cancer antigen specific T cell receptor (TCR) from one T cell to another [12], it is possible to produce large numbers of transgenic T cells for ACT. Autologous T cells from a patient could be transduced with cells from different patients with matching human leukocyte antigens (HLAs) [13].

Although ACT has shown promising results in some human malignancies, only a few solid tumor trials have demonstrated positive results because of numerous remaining problems [14]. These include tumor heterogeneity and antigen loss, hard trafficking and infiltration, immunosuppressive tumor microenvironment, etc. [15]. In this work, we will review recent advances and limitations in the clinical use of ACT and discuss approaches to improve its efficacy by addressing present hurdles.

## Types of adoptive cell therapy

### Tumor-infiltrating lymphocytes

There was evidence that immune infiltrates in the tumor microenvironment (TME) were crucial for tumor development and had a significant bearing on the clinical outcomes of those with cancers [16]. TILs in tumors were heterogeneous populations of cells that recognized a wide range of antigens [15]. By profiling TILs, we can gain insights into the mechanisms of cancer-immune evasion and develop new therapeutic strategies.

With the advent of ICIs and ACT, the role of T cells in antitumor immunity has become undeniable. T cells recognized antigens and mediated immune responses by TCRs. Immature T cells produce various cell clones carrying specific and diverse antigen TCR structures by genetic rearrangement and combination of a small number of germline gene segments, which can generate disparate TCR repertoires [17]. Positive selection endows mature T cells with the ability to recognize and bound major histocompatibility complex (MHC) or HLA, which present short peptides of tumor antigens to TCRs [18]. And then, negative selection eliminates T cells that show high affinity between their TCRs and self-MHC-peptide complexes [19]. T cells acquire functional, various TCRs and MHC restriction through these important events in development.

CD8^+^ T cells, also known as cytotoxic T lymphocytes (CTLs), are the critical effector cells responsible for killing tumor cells through two main mechanisms. First, activated CD8^+^ T cells directly and specifically destroy tumor cells by releasing granzymes (GZM) and perforin [20]. Second, they kill tumor cells indirectly by secreting cytokines, such as interferon-γ (IFN-γ), tumor necrosis factor-α (TNF-α) and lymphotoxin [21]. Almost all types of cancers benefit from CD8^+^ CTLs. However, tumor-infiltrating CTLs rarely control tumor growth due to exhaustion or dysfunction caused by immunosuppressive TME [22–24].

Assisting CTLs in overcoming negative regulation, CD4^+^ helper T cells enabled CTLs priming, as well as their effector and memory activities [25], which was found in a second T cell-priming step [26]. In the lymphoid organs, CD4^+^ and CD8^+^ T cells first interacted independently with conventional dendritic cells (cDCs), which was non-synchronous [27]. And then, on the same cDC, CD4^+^ and CD8^+^ T cells recognized their respective antigens, providing cytokines and co-stimulatory signals to promote proliferation and differentiation of CD8^+^ T cells [25]. To support the differentiation of CD8^+^ T cells into effector CTLs, cDCs produced Type I IFN, interleukin (IL)-12, and IL-15 [28, 29]. Another subset of CD4^+^ T cells could mediate cytotoxicity in cancers by expressing GZM and perforin, or directly exerting cytotoxic effects [30].

Recent studies have also shown that tumor-infiltrating B lymphocytes (TIL-Bs), which included tumor-infiltrating B cells and plasma cells, played a crucial role in immunotherapy. Their presence has been linked to improve prognoses for different cancer types, including breast cancer, head and neck squamous cell carcinoma lung adenocarcinoma, etc. [31–35]. Meanwhile, Catalina et al. demonstrated that B cells expressing PD-L1, CD155, IL-10 and tumor growth factor (TGF)-β could prevent activated CD8^+^ T cells from proliferating and obtaining an effector phenotype [36]. This was due to the different B cell phenotypes presented and the antibodies they produced, as well as the various composition of the TME [37]. TIL-Bs were capable of supporting antitumor immune responses in several ways. As antigen-presenting cells, B cells boosted cellular immunity by presenting tumor-associated antigens (TAAs) to T cells and facilitating the endocytosis by dendritic cells [38, 39]. Moreover, TIL-Bs were capable of killing tumor cells directly with cytokines like IFN-γ and GZMB [40]. Previous studies indicated that it was possible for plasma cells to produce IgG1 antibodies that could cause antibody-dependent cell cytotoxicity [41, 42]. Additionally, B cells were involved in the formation of tertiary lymphoid structures (TLSs) [43]. The prognostic value of TLSs has been demonstrated among the various types of cancer [44–46].

**Fig. 1:**
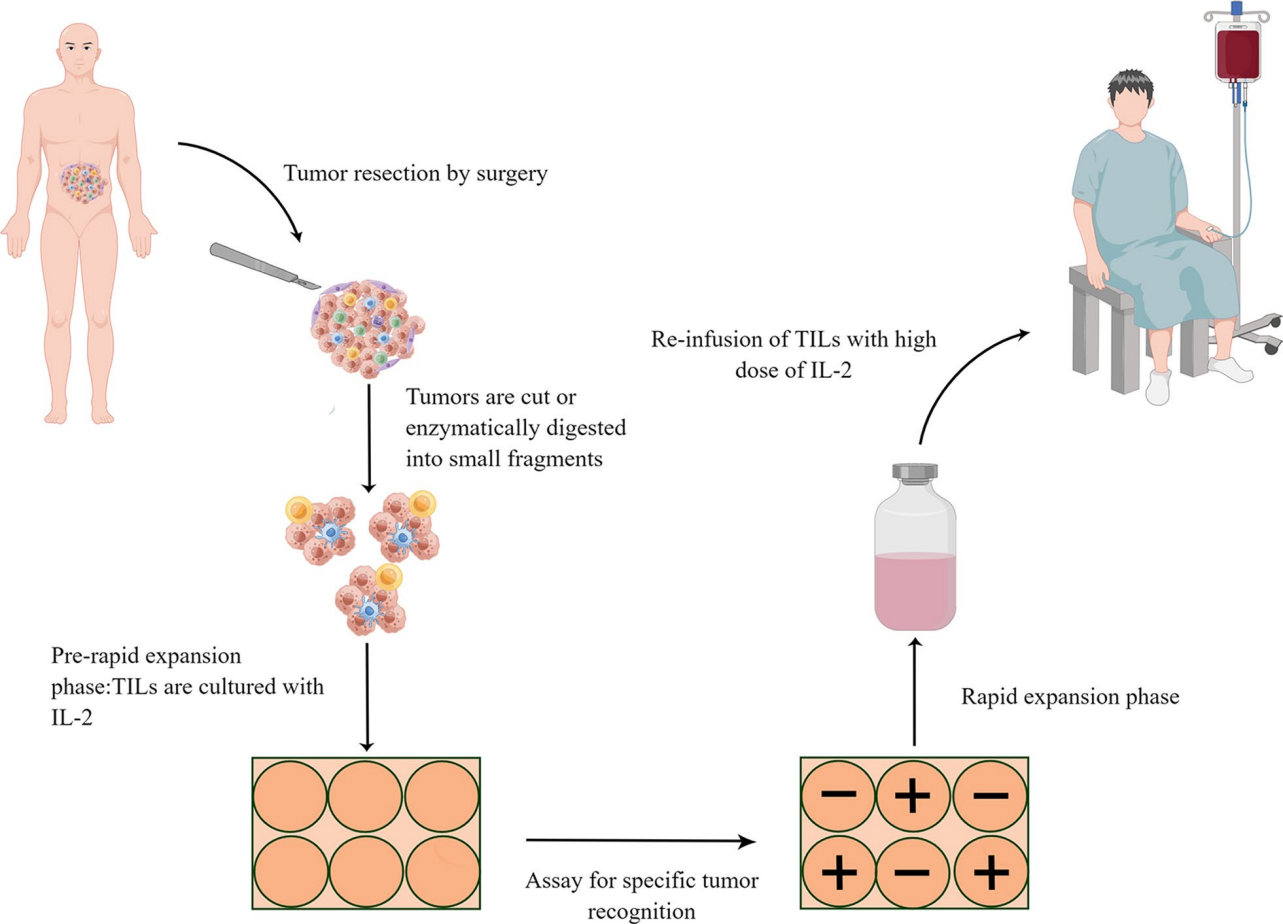
Process of TIL treatment. The tumor was excised by surgery and prepared into a single-cell suspension by mechanically cut and digestive enzymes. Different types of TILs proliferated on cell plates with high dose of IL-2, producing billions of TILs within three weeks. Any TILs that have anti-tumor effect were left as the positive TIL populations after culturing with the tumor cells of patients. TILs were then expanded to treatment levels by rapid expansion phase. Patients underwent lymphodepletion prior to receiving TILs, and then 10–150 billion TILs and high dose of IL-2 were administered into them. By Figdraw

The main process of TIL therapy included isolation of TILs from tumor tissue samples, amplification ex vivo and transfusion of TILs (Fig. 1). Researchers collected TILs from freshly resected melanomas and expanded ex vivo. Twenty patients with metastatic melanoma were treated with intravenous infusion of TILs, followed by injecting IL-2, which caused objective responses of eleven patients [47]. Since then, several studies have been conducted on TILs in cancer. In a phase II clinical trial, the autologous TILs were utilized to treat 93 patients with metastatic melanoma. This study found that adoptive transfer of TILs contributed to a 56% objective response rate (ORR) and a 22% complete response rate (CRR) [48]. Thirty-one metastatic melanoma patients who depleted their lymphocytes by using cyclophosphamide and fludarabine in advance, were treated with their harvested TILs and high-dose IL-2 [49]. The ORR was 48.4% and two patients had a complete response. Most studies on TIL therapy have focused on melanoma, although new data indicated TILs may also be effective in other solid tumors. Metastatic adenocarcinomas and squamous cell carcinomas associated with human papillomavirus were treated with TILs [50]. In this study, 7 of 29 (24%) patients had objective response and two of them had complete response. A clinical trial (NCT01174121) has demonstrated that adoptive transfer of TILs could mediate regression of metastatic colorectal cancer and breast cancer [51, 52]. It seemed that IL-2, fludarabine, and cyclophosphamide lymphodepletion were crucial for adoptive transfer TILs. It was not hard to see that recent studies have deliberately sought to identify which population of cells played a major role in adoptive transfer of TILs. Laszlo et al. found that CD8^+^ T cells expressing B and T-lymphocyte attenuator were strongly correlated with a beneficial clinical outcome [49]. Metastatic epithelial carcinoma could be reversed by CD4^+^ T lymphocytes that identified an erbb2 interacting protein mutation [53]. Complete cancer remission and T cell persistence were associated with stem-like neoantigen-specific CD8^+^ T cells [54]. Therefore, the best course of action could be to transfer TILs specifically for various tumor mutant neoantigens.

The role of B cells in adoptive transfer of TILs has also been described. It was reported that B cells gathered from tumor-draining lymph nodes, which were activated with LPS and anti-CD40 mAb, could inhibit spontaneous metastases of a 4T1 breast cancer model [55]. Yang et al. reported that the combination of activated B effector cells and IL-2 could kill 4T1 tumor cells by activating the CXCR4/CXCL12 and perforin pathways, as well as the Fas/FasL interaction [56]. 4-1BBL^+^ B cells activated with CD40 agonism and IFN-γ resulted in a powerful effect against glioblastoma cancer model [57].

In order to improve the effect of TIL products, combination approaches were being used, including the addition of checkpoint blockade. Patients with metastatic non-small cell lung cancer (NSCLC) were treated with TILs and nivolumab in a phase I trial (NCT03215810). In this study, Patients received 4 cycles of nivolumab prior to TILs infusion. Following lymphodepletion chemotherapy, TILs and IL-2 infusions were given to 16 patients who were proven progression. After that, patients continued receiving nivolumab every 4 weeks for up to a year. Of the 13 evaluable patients, three had proved to be responsive and two of them achieved a sustained complete response [58]. However, isolating tumor-specific TILs that were not present in all patients or expanding a small number of cells for therapeutic efficacy was sometimes problematic. On February 16, 2024, Lifileucel (Amtagvi), the first TIL therapy, was approved by the FDA for the treatment of advanced melanoma that has progressed after PD-1 antibody therapy.

### T cell receptor-transgenic T cells

TCRs are responsible for recognizing and binding antigens, which can activate T cells to participate in immune responses against infections and tumors. The transmission of signals to the interior of the T cell and subsequent activation occur exclusively upon the binding of the TCR to a specific MHC molecule, which is presenting a specific peptide chain. TCRs can be divided into two types which included TCRαβ and TCRγδ, and αβT cells account for the vast majority of total T cells and mediate cellular immunity. A functional receptor is formed by the complexing of TCR α/β heterodimers with CD3 ε/γ/δ/ζ subunits [59]. The CD3 cytoplasmic region is long and contains immunoreceptor tyrosine-based activation motif (ITAM) related to T cell activation signal transduction [60]. Activation of T cells and initiation of effector functions result from this process. The TCR-based therapy involves genetically modifying T cells from blood to express transgenic TCRs which are capable of recognizing tumor antigens and attacking cancer cells. Antigens produce or express abnormally by tumor cells in the course of cancerous and malignant growth are known as tumor antigens. The classes of tumor antigens that have been widely studied include TAAs and tumor-specific antigens (TSAs).

It is important to note that TAAs are expressed not only in tumor cells, but also in healthy tissues. Therefore, therapies targeting TAAs must take into consideration possible on-target toxicity caused by T cells. Moreover, proteins that are structurally similar to these antigens may also be targeted by T cells [61]. In several trials with different antigen targets, TCRs have been found to be clinically active against solid tumors (Table 1). Testes, embryos, and placentas all exhibited a subset of TAAs known as cancer testis antigens (CTAs) which started with the identification of the melanoma-associated antigen (MAGE) gene family [62]. As the cell dose-escalation phase progressed, an objective complete response occurred in a patient with metastatic cervical cancer treated with CD4^+^ T cells expressing TCRs that could recognize MAGE-A3 [63]. In a Phase I/II trial, HLA-DP0401/0402 restricted anti-MAGE-A3 TCR genetically modified cells and aldesleukin were administered after lymphatic clearance for metastatic malignancy expressing MAGE-A3 (NCT02111850). This resulted in partial responses in two patients and a complete response in one patient. Other clinical trials are actively underway, including TCRs targeting MAGEA1, MAGE-A3/A6, MAGEA4/8, MAGE-C2 (NCT03441100, NCT05430555, NCT03139370, NCT03247309, NCT04729543). Another CTA of high clinical significance was New York esophageal squamous cell carcinoma-1 (NY-ESO-1), which was highly expressed in various solid cancers and hematological tumors [64]. The effectiveness of transferring autologous NY-ESO-1-specific T cells was assessed in HLA-A*02 patients with synovial sarcoma (SS). This marked the first clinical study of designed TCR treatment for sarcoma, validating tumoral NY-ESO-1 expression [65]. Another group of SS patients with advanced HLA-A*02, separated into 4 groups based on tumoral NY-ESO-1 expression, were treated with the NY-ESO-1-targeting specific peptide enhanced affinity receptor (SPEAR) T cells in a pilot trial (NCT01343043). Across all cohorts, 35% (15/42) of patients experienced a full or partial response, with the remaining 57% (24/42) having stable disease [66]. The National Cancer Institute (NCI) treated 10 patients with high-dose aldesleukin and peripheral blood cells transduced with the anti-ESO murine TCR, achieving a 50% ORR and a 10% CRR (NCT01967823). Twenty-five patients with recurrent, refractory or high-risk multiple myeloma were enrolled in a phase II clinical trial group (NCT01352286). They assessed the function and safety of T cells with th e NY-ESO-1^c259^ TCR. At the end of the first year, 11/25 patients had responded after being infused a large number of NY-ESO-1 SPEAR T cells and no serious adverse events (AEs) and no cases of cytokine release syndrome (CRS) were recorded during this trial [67].

**Table 1:** Selected engineered TCR therapy trials in solid tumor from ClinicalTrials.gov

Target	Type of cancer	Phase	Clinical trial number
MAGEA1	Solid tumors	I	NCT03441100
MAGEA1	Advanced solid tumors	I/II	NCT05430555
MAGEA3	Cervical cancer; Renal cancer; Urothelial cancer; Melanoma; Breast cancer	I/II	NCT02111850
MAGE-A3/A6	Solid tumors	I	NCT03139370
MAGEA4/8	Solid tumors	I	NCT03247309
MAGE-C2	Melanoma; Head and neck Cancer	I/II	NCT04729543
NY-ESO-1	Neoplasms	I	NCT01343043
NY-ESO-1	Melanoma; Meningioma; Breast Cancer; Non-Small Cell Lung Cancer; Hepatocellular Cancer	II	NCT01967823
NY-ESO-1	Multiple Myeloma	II	NCT01352286
HPV-16 E7	Metastatic or refractory/recurrent human papillomavirus (HPV)-16 + cancers	I/II	NCT02858310
HBV	Hepatocellular Carcinoma	I	NCT03899415
EBV	Nasopharyngeal Carcinoma	III	NCT02578641
EBV	Nasopharyngeal Carcinoma	I/II	NCT04509726

The combination of engineered TCR therapy with genome editing offered the potential to improve the efficacy and safety of modified T cells. Edward et al. conducted a phase I human pioneer study. They targeted endogenous T cells using CRISPR-Cas9 technology to knock out TCR α chain gene, TCR β chain gene, and PD-1 gene, thereby increasing the activity and safety of NY-ESO-1 TCR-engineered T cells and demonstrating initial feasibility [68]. However, only a restricted number of patients might benefit from these medicines because of the limited cancer types that expressed these TAAs and the limitation of HLA types. Furthermore, the heterogeneous expression of TAAs on malignancies complicated this therapy [69].

Due to TSAs are expressed only on tumor cells, T cell receptor-transgenic T (TCR-T) exerts a stable anti-tumor effect without harming normal cells [70]. TSAs currently under investigation included viral antigens and neoantigens. Viral antigens were mainly referred to those antigens that were associated with tumorigenesis. Viral infections were the root cause of many human cancers. When it comes to human papillomavirus (HPV), we considered it to be primarily related to cervical cancer [71]. The involvement of HPV in the development of various malignancies, such as vaginal, vulvar, anal and oropharyngeal cancers have lately been studied [72–74]. Hepatitis B virus (HBV) is a non-cytopathic DNA virus that could induce chronic infection, eventually leading to hepatocellular cancer [75]. Epstein-Barr virus (EBV) is a ubiquitous, oncogenic virus that was linked to a variety of human cancers, including nasopharyngeal carcinoma (NPC), Hodgkin lymphomas, non-Hodgkin lymphomas (NHL), natural killer (NK)/T cell lymphomas and a subset of gastric cancers [76, 77].

In a clinical trial with a primary endpoint of maximum tolerated dose, 6 of 12 patients treated with HPV-16 E7 TCR-T cells showed objective clinical responses (NCT02858310). Cancer regression was considerable in several individuals, with some tumors regressing durably [78]. For example, after 8 months of treatment, 31% of complete tumor regression occurred in one patient with more than 80 metastatic tumors. No treatment-related damage to normal tissue or deaths were found in this study. Preliminary evidences of anticancer efficacy and safety have been obtained from an ongoing phase I clinical trial on eight patients with advanced hepatocellular carcinoma employing short-lived HBV-specific TCR-T cells [79]. For EBV, a completed phase III clinical trial combining gemcitabine and carboplatin with EBV-specific T cells for NPC resulted in a 9% CRR and a 63% ORR (NCT02578641). Moreover, LMP2-specific TCR-T cells with IL12 auto-secreting element have been used to treat NPC in a clinical trial (NCT04509726). However, cancers caused by viruses accounted for only a portion of cancers [80], and the effective application of TCR-T cells may depend heavily on the discovery of neoantigens.

Neoantigens were mutated peptides originating from somatic mutations that were absent from normal tissues and specifically recognized by TCR-T cells [81]. The TCRs had the precise specificity to recognize neoantigens generated by single point mutations in peptide sequences presented by the MHC on the surface of cancer cells. Therefore, they were ideal targets for engineered TCR therapy. For the development of cell-based cancer immunotherapies, the precise identification of antitumor TCRs posed1 a significant hurdle. Single-cell RNA sequencing, T cell receptor sequencing, whole-exome/transcriptome sequencing, mass spectrometry and NeoScreen were used to discover T cells that specifically recognized neoantigens [82–84]. Current immunotherapeutic strategies against neoantigens often target patient-specific private antigens produced by non-recurrent driver mutations or passenger mutations [85]. Identification of neoantigens will allow engineered TCR therapy to become more individualized. In this phase 1 clinical trial (NCT03970382), researchers employed whole genome sequencing to identify patient-specific tumor mutations, while RNA sequencing was utilized to discern expression levels of genes associated with these mutations. Consent was obtained from patients who contributed tumor biopsies and peripheral blood mononuclear cells for the screening of personalized neoTCR products. In order to concurrently knock out endogenous TCRs and knock in TCRs that specifically target patient-specific neoantigens using non-viral precision gene editing, several TCRs that detect patient-specific mutations in neoantigens have to be identified and generated [86]. There are still ongoing investigations using autologous T cells modified to express TCRs reactive against neoantigens in patients with refractory solid malignancies (NCT05194735, NCT04520711, NCT05349890, NCT03412877).

However, the produce of personalized TCR-T cells for each patient was time-consuming and costly, which increased the possibility of disease progression. Therefore, targeting a public neoantigen that arose from a driver hotspot mutation and was presented by a common HLA allele may circumvent many of the limitations. For instances, a single infusion of autologous T cells was given to a patient with metastatic pancreatic adenocarcinoma who had not responded to TIL therapy, causing a 72% overall partial response. To specifically target KRAS G12D, two allogeneic TCRs that were HLA-C*08:02-restricted were expressed on these T cells through genetic modification [87]. However, another patient died after 6 months of treatment, even though he had a high levels of specific T cells in his blood and no mechanisms of immunotherapy resistance were found. In a study initiated by NCI (NCT00068003), 97 patients with chemorefractory metastatic epithelial cancer were shown to contain nonsynonymous *TP53* mutations [88]. Smita S et al. demonstrated that mutant *PIK3CA* produced an immunogenic public neoantigen shared among HLA-A*03:01^+^ patients [89]. However, there were few public neoantigens available for targeting, which may lead to treatment resistance due to antigen loss [90]. Due to the absence of neoantigens presented by common HLAs resulting from specific driver gene mutations, the actual cohort of patients who could benefit from public neoantigen-targeted therapies was notably smaller than the projected theoretical number. We also needed to know whether the public neoantigen was immunogenic for TCR-T cell therapy.

### Chimeric antigen receptor T cells

**Fig. 2:**
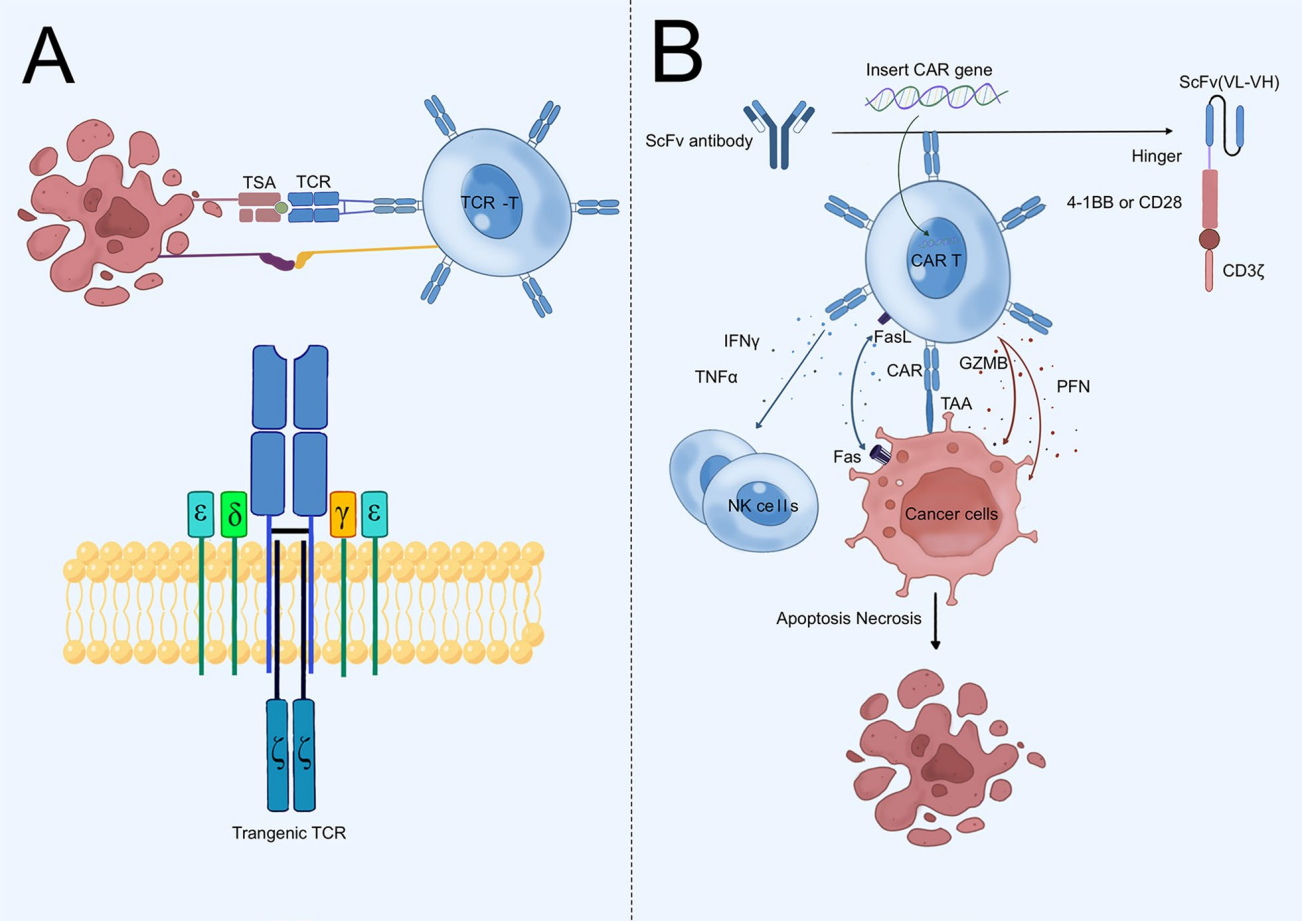
Differences between T cell receptor-transgenic T (TCR-T) cells and chimeric antigen receptor (CAR) T cells. **A** Transgenic TCRs were capable of forming functional TCR-CD3 complexes and did not differ from the standard TCR structure of an α/β chain heterodimer. Major histocompatibility complex (MHC)-presented intracellular peptide antigens were recognized by TCRs. The two intracellular CD3 domains triggered downstream TCR signaling upon antigen recognition. **B** CARs, unlike TCRs, were unable to assemble CD3 complexes, and the single-chain fragment variable (scFv) did not require MHC to recognize surface antigens. By Figdraw

TCRs were able to recognize antigens with the restriction of MHC, therefore, T cells with particular TCR could only be utilized to treat patients with the corresponding MHC genetic background, limiting the usage of TCR-T. CAR is a modular, genetically modified synthetic antigen receptor with antibody-like characteristics and efficient TCR activation signaling [91]. In contrast to TCR-T cells, CAR T cells can kill tumor cells without the restriction of MHC (Fig. 2).

The first generation of CAR T cells incorporated a single-chain fragment variable (scFv) to the CD3ζ signaling chain, which included the ITAM, endowing T cells with MHC-independent activation [92]. However, these CAR T cells have demonstrated absolutely no efficacy. To improve cell proliferation and cytotoxicity, the second generation of CAR incorporated a co-stimulatory structural domain mostly derived from CD28 or 4-1BB (CD137), located between the transmembrane and CD3 signaling regions [93]. Compared with CD28-based CARs, which induced effector-like memory cell differentiation and secreted higher levels of cytokines, CARs with 4-1BB could increase central memory T cell differentiation and persistence, as well as enhance mitochondrial biogenesis with increased fatty acid metabolism [94]. The design of the third generation of CAR combined the different advantages of the two co-stimulatory structural domains to further enhance the activity of CAR T cells. The fourth-generation CAR-T cells, also known as T cells redirect for universal cytokine killings (TRUCKs) were designed to release cytokines into tumor tissues when the CARs bound to targeted antigens. The cytokine which was overexpressed in CAR was IL-12, a highly effective substance that used NK cells to destroy tumor cells that were not identified by CARs and boosted the IFN-γ, GZMB and perforin secretion of T cells [95]. An IL-2 receptor domain between the CD3 and CD28 signaling domains as well as a STAT3-binding motif were recently added to a unique CAR that followed the framework of the second generation [96]. Until now, seven CAR T cells have been approved for marketing worldwide (Table 2).

**Table 2:** CAR T therapeutic products approved for commercial use

Trade name	Co-stimulatory domain	Target	Indication	Dose level (cells)	Clinical efficacy	Clinical Trial
Kymriah	4-1BB	CD19	R/R ALL (≤ 25 years of age)	3.1 × 10^6^/kg	ORR: 81% CRR: 60%	NCT02435849
			R/R LBCL	3 × 10^8^	ORR: 52% CRR: 40%	NCT02445248
			R/R FL	2.06 × 10^8^	ORR: 86% CRR: 69%	NCT03568461
Yescarta	CD28	CD19	R/R LBCL	2 × 10^6^/kg	ORR: 82% CRR: 54%	NCT02348216
			R/R FL	2 × 10^6^/kg	ORR: 94% CRR: 79%	NCT03105336
Tecartus	CD28	CD19	MCL	2 × 10^6^/kg	ORR: 91% CRR: 68%	NCT02601313
			ALL	1 × 10^6^/kg	ORR: NR CRR: 71%	NCT02614066
Breyanzi	4-1BB	CD19	LBCL	50 × 10^6^ 100 × 10^6^ 150 × 10^6^	ORR:68% CRR:60% ORR:74% CRR:52% ORR:73% CRR:51%	NCT02631044
Abecma	4-1BB	BCMA	R/R MM	150 × 10^6^ 300 × 10^6^ 450 × 10^6^	ORR:50% CRR:25% ORR:69% CRR:29% ORR:81% CRR:39%	NCT03361748
Carvykti	4-1BB	BCMA	R/R MM	0.75 × 10^6^/kg	ORR:98% CRR:83%	NCT03548207
JWCAR029	4-1BB	CD19	R/R LBCL	100 × 10^6^ or 150 × 10^6^	ORR:76% CRR:52%	NCT04089215

R/R, relapsed/refractory; ALL, acute lymphoblastic leukemia; LBCL, large B-cell lymphoma; FL, follicular lymphoma; MCL, mantle cell lymphoma; MM, multiple myeloma; ORR, objective response rate; CRR, complete response rate; NR, not reported

An autologous CD19-targeted CAR T cell therapy called Tisagenlecleucel (Kymriah) was first approved for the treatment of patients under the age of 25 with relapsed/refractory (R/R) B-cell precursor acute lymphoblastic leukemia (B-ALL) based on a global trial (NCT02435849) [97]. Tisagenlecleucel was latter authorized for the treatment of adult patients with R/R large B-cell lymphoma (LBCL) [98] Recently, Tisagenlecleucel was allowed for R/R follicular lymphoma (FL) based on the ELARA phase II global trial (NCT03568461). In this trial, Tisagenlecleucel was administered in 97 enrolled patients with R/R FL (below grade 3B). The ORR was 86% and the CRR was 69% of the 94 patients who could be evaluated for efficacy, and 46% of the patients developed Tisagenlecleucel-related AEs of grade 3 or 4 [99].

In October 2017, the FDA approved Axicabtagene ciloleucel (Yescarta) for the treatment of adult patients with R/R LBCL following two or more lines of systemic therapy. This included patients with diffuse large B-cell lymphoma (DLBCL), primary mediastinal large B-cell lymphoma (PMBCL), high-grade B-cell lymphoma (HGBCL) and DLBCL arising from FL [100]. In a pivotal phase I/II trial called ZUMA-1 (NCT02348216), the ORR and CRR were 82% and 54%, respectively [101]. Grade 3 or higher CRS, as well as immune effector cell-associated neurotoxicity syndrome (ICANS), affected 13% and 28% of patients, respectively. ZUMA-5 was a single-arm and multicentre phase II trial (NCT03105336), which the overall response was achieved in 94% of the 84 FL patients, with a complete response in 79% of those patients [102], causing the approval of the indication for adult patients with R/R FL following two or more lines of systemic treatment. In an international phase III trial, ZUMA-7 (NCT03391466), Yescarta demonstrated a higher event-free survival and response rate in patients with early R/R LBCL compared with second-line standard care [103]. A retrospective study found that R/R LBCL patients administrated with Axicabtagene ciloleucel had higher toxicity but comparable non-relapse mortality and efficacy to those treated with tisagenlecleucel [104].

Brexucabtagene autoleucel (Tecartus) was the sole CAR T cell therapy recognized for the treatment of individuals with mantle cell lymphoma (MCL), which targeted CD19 [105]. After a 3-year follow-up in the key ZUMA-2 study (NCT02601313), 68 patients with R/R MCL received Brexucabtafene autoleucel infusion still showed an ORR as high as 91% (68% CRR) [106]. With a median duration of response (DOR) of 28.2 months, responses were long-lasting, and 37% of the patients who underwent treatment still had positive effects. In the retrospective historical external control study SCHOLAR-3, OS of patients with R/R B-ALL in the ZUMA-3 clinical trial (NCT02614066) was nearly 20 months longer than matched patients who received standard-of-care therapies [107].

Lisocabtagene maraleucel (Breyanzi) was a CD19-directed CAR T cell product with a 4-1BB co-stimulatory domain that consisted of purified CD8^+^ and CD4^+^ CAR T cells in a specific ratio (1:1) [108]. Based on an ORR of 73% and a CRR of 53% in 256 patients in the TRANSCEND NHL 001 trial (NCT02631044), Lisocabtagene maraleucel was approved for R/R LBCL following two or more systems of therapy, including DLBCL, HGBCL, PMBCL and FL grade 3B [109]. In TRANSFORM (NCT03575351) phase III trial, 184 patients with LBCL who were primary refractory or relapsed within 12 months of the first-line therapy were randomly assigned to one of two treatment groups: Lisocabtagene maraleucel or standard of care [110]. When compared to standard-of-care treatment, treatment with Lisocabtagene maraleucel increased median event-free survival by over 8 months and demonstrated a higher CRR (66% vs 39%) [110]. ICANS (Grade3 = 4%) and CRS (Grade3 = 1%) were occasionally reported.

Idecabtagene vicleucel (Abecma) became the first CAR T therapy approved for the treatment of adult patients with R/R multiple myeloma (MM) who have received ≥ 4 lines of prior therapies on March 26, 2021 [111]. Idecabtagene vicleucel was a genetically modified autologous CAR T cell therapy with a 4-1BB co-stimulatory domain, targeting B-cell maturation antigen (BCMA) [112]. The type III transmembrane protein BCMA, also known as CD269 or TNFRSF17, was expressed only by normal and malignant plasma cells, constituting a member of the TNF receptor superfamily. [113]. In the KarMMa trial (NCT03361748), patients with R/R MM who had undergone at least three prior treatments were administrated Idecabtagene vicleucel target doses ranging from 150 × 10^6^ to 450 × 10^6^ CAR T cells [114]. The best outcomes were shown in patients who received 450 × 10^6^ dose, with 81% of them demonstrating an objective response and 39% demonstrating a complete response [114]. Compared to other CAR T products, the CRR of Abecma needed further improvement.

The second CAR T therapy to target BCMA was Ciltacabtagene autoleucel (Carvykti, LCAR-B38M, JNJ-4528), which was used to treat R/R MM in adults. This was the first Chinese CAR T cell treatment to be approved by the FDA. Two BCMA-targeting domains on the Ciltacabtagene autoleucel have been designed to increase avidity [115]. LCAR-B38M demonstrated potent efficacy in the LEGEND-2 longest follow-up (NCT03090659), with an ORR of 87.8% (73% CRR), minimal residual disease negativity rate of 67.6%, median DOR of 23 months and median progression-free survival (PFS) of 18 months in R/R MM after 4 years [116]. A phase Ib/II study called CARTITUDE-1 (NCT03548207) was conducted on Ciltacabtagene autoleucel with the goal of evaluating the drug efficacy and safety [117]. The ORR for CARTITUDE-1 was 97.9% after a 2-year follow-up, with 82.5% of patients achieving a stringent complete response [118]. The median PFS and OS were not attained. Only 4% of CRS patients had a grade 3 or higher, with grade 1 or 2 individuals making up the majority [119]. Neurotoxicity events, including ICANS and other neurotoxicities, occurred in 20 individuals following Ciltacabtagene autoleucel infusion [120].

Relmacabagene autolucel (JWCAR029), an autologous CAR-T cellular immunotherapy product that targeted CD19, was approved by the National Medicine Products Administration (NMPA) for the treatment of adult patients with R/R LBCL following the second-line or more systemic therapies. When compared to Lisocabtagene maraleucel, Relmacabagene autolucel used microbeads to provide a variety of dosages with similar product qualities without the requirement for distinct CD4 and CD8 T-cell production trains [121]. The best ORR and CRR in 58 patients with evaluable efficacy in the pivotal phase II clinical study (NCT04089215) were 75.9% and 51.7%, respectively [121]. On the safety side, the percentage of grade 3 or higher CRS and neurotoxic events in 59 treated patients were less than 5%.

In addition to its application in oncology, CAR T therapy has shown significant promise for the treatment of many types of autoimmune diseases. CAR T cell can treat many different types of autoimmune diseases by targeting and eliminating pathogenic immune cells implicated in the disease pathology. In addition, CAR regulatory T cell (Treg) therapy activates and proliferates Treg cells, which further enhances the immunosuppressive effect. Mycophenolate mofetil, an immunosuppressant, in conjunction with CD19-targeting CAR T cells had the potential to disrupt pathogenic B-cell and T-cell responses, leading to remission in patients with refractory antisynthetase syndrome [122]. Anti-CD19 CAR T cells were proven to lead to deep B-cell depletion, clinical symptoms improvement, and drug-free remission in five patients with systemic lupus erythematosus [123]. A recent study demonstrated favorable therapeutic outcomes and controllable safety of anti-CD19 CAR T cells in patients with severe systemic lupus erythematosus, idiopathic inflammatory myositis, and systemic sclerosis [124]. CAR Treg has also shown potential in preclinical studies and early clinical trials for a variety of autoimmune diseases, including graft-versus-host disease, type 1 diabetes, multiple sclerosis, etc. For instance, in immunodeficient mice that were reconstituted with human PBMCs, anti-CD19 CAR Tregs inhibited the generation of antibodies, lowering the likelihood of graft-versus-host disease [125].

## Challenges and strategies of ACT in solid tumors

### Tumor heterogeneity and antigen loss

The success of ACT in hematological malignancies was attributable to the specific expression of identifiable antigens (CD19 and BCMA) on tumors. However, only a small number of antigens were tumor-specific. The majority of candidate antigens frequently co-expressed on normal and cancerous tissues, posing a significant risk of morbidities due to on-target, off-tumor toxicity [126]. Some tumor-specific neoantigens have been found thanks to the advancement of genomic and proteomic methods. Disialoganglioside (GD2) was overexpressed in some tumor types but showed limited expression in normal tissues [127]. In a phase I clinical trial (NCT04196413), 75% of patients with H3K27M-mutated diffuse intrinsic pontine glioma (DIPG) or spinal cord diffuse midline gliomas, exhibiting high GD2 expression, experienced clinical and radiographic improvements. No on-target or off-tumor toxicity was observed [128].

Despite the fact that some antigens may be overexpressed on tumor cells, solid tumors had a high degree of heterogeneity, which was a common mechanism of therapy resistance [129]. Based on a phase III trial (NCT03070392), Tebentafusp was approved by the FDA on January 25, 2022, for the treatment of people with HLA-A*02:01-positive metastatic or incurable uveal melanoma [130]. Compared to the control group, 252 patients with metastatic uveal melanoma who received tebentafusp treatment had longer OS (73% vs 59%) [131]. The immune-mobilizing monoclonal TCR against cancer, in which the anti-CD3 scFv was capable of recruiting any T cells to the tumor cell perimeter, was the foundational component of tebentafusp. This method was independent of the tumor mutation status or the presence of tumor antigen-specific T cells. High antigen density requirements were present in traditional CAR designs, but the antigen density thresholds could be tuned by optimizing the CAR designs [132]. The expression of Glypican 2 (GPC2) was higher in optic neuroblastoma tissues compared to normal pediatric tissues, making it a prime candidate for CAR T cell therapy [133]. The GPC2-CAR T cells, equipped with CD28TM/endodomains and augmented c-Jun expression, effectively lowered the threshold of GPC2-CAR antigen density. This enhancement facilitated the proficient and sustained eradication of neuroblastomas exhibiting clinically relevant GPC2 antigen densities [134]. One of the most clearly reasons for relapse following CAR T cell therapy was antigen loss. To overcome antigen loss and lower the likelihood of tumors resistance, CARs against multiple antigens have been tested. CD20 and CD22 were promising CD19-negative tumor targets for CAR T cell therapy [135]. In a phase I/II single-arm trial (NCT03097770), CD19/CD20 CAR T cells were administered to 87 patients with R/R NHL. The best ORR among 87 patients was 78%, with 70% of patients achieving a complete response [136]. The bispecific CARs were effective in seven of nine patients who had relapsed after receiving CD19-CAR T cells, and only one of sixteen patients who relapsed after treatment of CD19/CD20 CAR T cells was found to have antigen loss [136]. The effectiveness and safety of bispecific CARs, as well as their capacity to prevent antigen loss in lymphoma patients, have been confirmed by additional clinical trials [137–142]. Table 3 summarized the multi-antigen-targeting CAR T cells for B cell malignancies being studied. The similar approach has also been conducted for other cancers, including glioblastoma [143], lung cancer [144], cholangiocarcinoma [145], gastric cancer [146] and hepatocellular carcinoma [147]. These findings suggested that multispecific CAR T cell therapy could be a promising strategy for preventing relapse due to antigen loss. However, a tiny proportion of individuals had antigen loss. Furthermore, more clinical trials and longer follow-up were required to assess the safety of multispecific CAR T cell therapy, particularly for on-target, off-tumor toxicity [148].

**Table 3:** Selected multi-antigen-targeting CAR T cell therapy

Target	Type of cancer	Dose level (cells per kg)	Response	Antigen loss relapse	Clinical trial
CD19/CD20	R/R B-NHL and CLL	2.5 × 10^5^–2.5 × 10^6^	ORR: 82% CRR: 64%	1/13 CD20 loss	NCT03019055
CD19/CD20	R/R NHL	0.5–8 × 10^6^	ORR: 78% CRR: 70%	1/16 CD19/CD20 loss	NCT03097770
CD19/CD22	R/R aggressive B-cell lymphoma	4.9–9.4 × 10^6^	ORR: 87.5% CRR: 62.5%	None	ChiCTR1800015575
CD19/CD22	R/R B-ALL and LBCL	1–3 × 10^6^	ORR: 79% CRR: 55%	9/24 CD19 loss/low	NCT03233854
CD19/CD22	R/R B-ALL and B-NHL	CD19: 2.6 ± 1.5 × 10^6^/5.1 ± 2.1 × 10^6^ CD22: 2.7 ± 1.2 × 10^6^/5.3 ± 2.4 × 10^6^	ORR: 89% CRR: 77%	1/42 CD19 loss	ChiCTR-OPN-16008526
CD19/CD22	R/R B-NHL	CD19: 4.1 × 10^6^ CD22: 4.0 × 10^6^	ORR: 90.5% CRR: 81%	None	ChiCTR-OPN-16009847
CD19/CD22	R/R B-ALL	1.7 × 10^6^–3 × 10^6^	CRR: 100%	1/3 CD19/CD20 loss/low	NCT03185494

R/R, relapsed/refractory; B-NHL, B cell non-Hodgkin lymphoma; CLL, chronic lymphocytic leukemia; B-ALL, B cell acute lymphoblastic leukemia; LBCL, large B-cell lymphoma; ORR, objective response rate; CRR, complete response rate

### T-cell trafficking and infiltration

Anti-tumor efficacy needs effective trafficking of effector cells to tumor tissues, which depend on the interaction between chemokines released by tumor cells and chemokine receptors on T cells [149]. Given that larger and persistent CAR T cells were associated with a higher likelihood of response in the blood of lymphoma patients, the difficulty of ACT to traffic to tumor sites due to disruption of the chemokine axes helps explain the lack of efficacy of these therapies in solid tumors to date. Tumors presumably diminished or even silenced the activity of chemokine axes involved in anti-tumor responses, while likely increasing the activity of chemokine axes engaged in pro-tumor immune cell activation [150]. In NSCLC, co-expression of tumor antigen mesothelial protein specific CAR T cells with the chemokine receptor CCR4 increased the migration of CAR T cells [151]. The infiltration of CAR T cells into glioblastoma was facilitated by CXCL11-armed oncolytic adenovirus, which also reprogramed the immunosuppressive TME to produce a significant antitumor effect and prolong survival [152]. When administered without prior lymphodepletion, CAR T cells were potentially modified to generate chemokine ligands like CCL19 to promote the recruitment of endogenous T cells and dendritic cells to tumor locations [153].

T cells encountered strong physical obstacles that could prevent their infiltration and impair their activity as they trafficked to solid tumor locations. The tumor stroma, which was made up of extracellular matrix (ECM), cancer-associated fibroblasts (CAFs), and the aberrant vasculature at the tumor site is a major hurdles [154]. To overcome these issues, a variety of techniques to increase ACT infiltration have been proposed. Mild heating has been proven to increase the infiltration of transferred cells by directly killing tumor cells and partially destroying ECM when combined with ACT [155]. Others have concentrated their efforts on matrix-degrading enzymes such as collagenases and hyaluronidases. Nearly all ECM components could be degraded by matrix metalloproteinases (MMPs), a class of calcium and zinc-dependent proteolytic enzymes [156]. MMPs could be secreted by macrophages [157]. A breast cancer study revealed that CAR-147 macrophages reduced collagen deposition in tumors and enhanced T cell infiltration into tumors, leading to a suppression of human epidermal growth factor receptor 2 (HER2)-4T1 tumor growth in mice [158]. The ECM was disrupted and more endogenous CD8^+^ T lymphocytes could be produced by targeting CAR T cells to fibroblast activating proteins (FAP) that were present in the stroma of most cancers [159]. Furthermore, a study found that modifying CAR-T cells to secrete heparinase enzyme could destroy the tumor matrix and improve tumor infiltration while increasing anticancer efficacy [160]. Fibronectin, an essential scaffold of the ECM, acted as a barrier between T cells and tumor cells, significantly affecting T cell infiltration [161]. Extra domain A (EDA) and extra domain B (EDB), two selectively spliced fibronectin exons, are overexpressed in most cancers but barely expressed in healthy tissues [162]. In vitro, Anti-EDA EDA CAR-T cells recognized and eliminated tumor cell lines that expressed EDA, and they exhibited antitumor effects in mice with immunocompetence [163]. In addition, when NSG mice were exposed to the human hepatocarcinoma cell line PLC, the human version of EDA CAR, which contained the human 4-1BB and CD3 endo domains, demonstrated potent antitumor activity [163]. EDB-CAR T cells had powerful anti-tumor activity in three xenograft mouse models and behaved in an antigen-dependent manner in vitro while posing minimal on-target, off-tumor toxicity [164]. Integrin αvβ3, a crucial component of tumor angiogenesis and metastasis, is typically promoted by the hypoxic TME. Therefore, it is a desirable target for ACT therapy [165]. In NSG mice, αvβ3 CAR-T cells significantly suppressed DIPG and glioblastoma and showed durable efficacy [166].

The structure and function of tumor blood vessels are markedly different from those of normal blood vessels, exhibiting unusual leakiness, high tortuosity, and inadequate pericyte coverage being some of their distinguishing features [167]. The delivery of T cells to tumors was hampered by aberrant vasculature, which also created an immune-hostile microenvironment and hypoxic tumors [168]. The initial solutions focused on anti-angiogenesis, a therapy that deprived tumors of oxygen and nutrients, resulting in delayed tumor growth [169]. However, since numerous growth factors, as well as a wide variety of cytokines and biomolecules, are involved in the process of angiogenesis, the current anti-angiogenesis therapies, which primarily target vascular endothelial growth factor (VEGF), have only a temporary effect on tumors [170]. Furthermore, destroying tumor vasculature may foster tumor metastasis with little reward to patient survival [171]. As a result, vasculature normalization was becoming more popular as an effective method. Vasculature normalization improved the efficiency of anti-tumor therapy and prevented tumor metastasis by restoring the structure and functionality of tumor vessels within a specific time window [172]. Tumor vasculature served as an effective target for CAR T cells due to its stable expression. Vascular endothelial growth factor receptor (VEGFR)-2 CAR T cells were more effective at controlling tumors when an anti-VEGF-A antibody was also administered in B16 tumor-bearing mice [173]. A recent study showed that the inhibition for phosphoglycerate dehydrogenase improved T cell infiltration and activation in tumors, pruned the aberrant vasculature, and made glioblastoma more responsive to CAR T immunotherapy [174].

Other strategies have also been used to enhance T cell infiltration. For instance, Tao et al. infused tumor-specific targeting peptides identified by phage display biopanning technology onto the T cell membranes to precisely target highly heterogeneous solid tumors while also significantly increasing CD8^+^ T cell infiltration [175]. Interestingly, scientists found that glucose/mannose analogue 2-deoxy-D-glucose, a medication that prevented N-glycan synthesis, eliminated the protective effect of N-glycans on tumors and made solid tumors more accessible for CAR T cells to infiltrate and eradicate [176].

### Immunosuppressive tumor microenvironment

Effective tumor-specific T cell responses to cancers were severely hampered by the immunosuppressive TME. Effector cells in immunosuppressive TME could be inhibited by immunosuppressive cells, immune inhibitory ligands on the surface of tumor cells and immunosuppressive cytokines [177].

#### Targeting immunosuppressive cells

Tregs, tumor-associated macrophages (TAM), and myeloid-derived suppressor cells (MDSC) are major immunosuppressive cells [178]. Immunosuppressive cells diminish the efficacy of ACT through multiple mechanisms. For instance, Treg suppressed or killed effector T cells by secreting cytotoxic substances like GZMB and immunosuppressive cytokines like TGF-β and IL-10 [179]. Treg also depleted the T-cell growth factor IL-2 by expressing a high level of CD25, a subunit of the IL-2 receptor, thereby limiting T-cell activation and proliferation [180]. Another strategy used by Treg to suppress T cells was the upregulation of immune checkpoint molecules such as lymphocyte activation gene 3 (LAG-3), CTLA-4, PD-1, and inducible co-stimulatory factor (ICOS) [181]. Basic researches have utilized a number of strategies to target immunosuppressive cells in order to improve the capacity of CAR T cells to eradicate tumors. When administered intratumorally, IL-12 has been demonstrated to improve CAR T cells cytotoxicity targeting epidermal growth factor receptor variant III and decreased the number of Tregs in a mouse model of orthotopic glioblastoma multiforme [182]. When the tumor necrosis factor–related apoptosis-inducing ligand receptor 2 and 4-1BB receptor were co-expressed on CAR T cells, the CAR T cell responses against tumor-associated mucin 1 or HER2 on breast cancer were improved. This resulted in TME remodeling and enhanced T cell proliferation at the tumor site [183]. CAR T cells that targeted folate receptor β, a characteristic of immunosuppressive M2 TAM cells, increased CD8^+^ T cell infiltration and enhanced the efficacy of CAR T cells in ovarian cancer, colon cancer, and melanoma mouse models [184]. Together, these developments potentially ushered in a new era for CAR T cell treatment by converting immunosuppressive cells to a more advantageous niche.

#### Targeting immune checkpoints

Immune checkpoint receptors and ligands, including PD-1, PD-L1, CTLA-4, LAG-3, T cell immunoglobulin and mucin-domain containing-3, B7-H3 and V-domain Ig suppressor of T cell activation, have been shown to impair the efficacy of ACT and produce anergy in TME [185]. Therapies targeting PD-1 have been utilized in conjunction with ACT to improve the lifespan and effectiveness of CAR T cells. Disruption of PD-1 by CRISPR-Cas9 increased cytokine output and cytotoxicity of CAR T cells against PD-L1^+^ cancer cells without reducing proliferation [186]. Researchers discovered that co-expression of a PD-1 decoy receptor may be able to bypass the inhibitory signaling of B7-H1/PD-1 in the TME of solid tumors and dramatically increase the therapeutic efficacy of B7-H3 specific CAR T cells [187]. Others have developed CAR T cells to release bispecific trap proteins that specifically target PD-1 and TGF-β, attenuating suppressive T cell signaling, boosting T cell persistence and proliferation, and promoting effector function and resistance to exhaustion [188]. Although this targeting technique has not been thoroughly researched, similar strategies could be utilized against other immune checkpoints. B7-H3, also known as CD276, has been implicated in tumor growth, metastasis, and treatment resistance, all of which contribute to a bad prognosis for patients by assisting cancer cells in evading the surveillance by cytotoxic T cells and NK cells [189]. In a first-in-human phase I trial (NCT04185038), B7-H3 CAR T cells were administered to children with R/R central nervous system malignancies and DIPG, which demonstrated correlated evidence of local immune activation and persistent cerebrospinal fluid B7-H3 CAR T cells [190]. In fact, checkpoint blockade could effectively fuel CARs and enhance the efficacy of CAR T cells.

#### Targeting cytokines

Although activated T cells secreted a variety of cytokines, including IFN-γ and IL-2, these were obviously insufficient to maintain the prolonged effects of T cells against tumors. In preclinical and clinical trials, many cytokines have been employed in conjunction with CAR T cells to improve the efficacy of ACT [191]. In a disseminated, syngeneic RM9-hSTEAP1 tumor model in hSTEAP1-KI mice, STEAP1 CAR T cells combined with a collagen binding domain IL-12 enhanced OS, cytokine production, tumor antigen presentation, and dissemination with epitopes to prevent STEAP1 antigen escape [192]. As a cytokine involved in T cell activation, maintenance, and proliferation, IL-7 enhanced CAR T cell effectiveness in vivo and facilitated tumor cell killing [193]. In addition to increasing the number of CAR T cells, recombinant human IL-7 fused with hybrid Fc (rhIL-7-hyFc) significantly enhanced T cells cytotoxicity and lowered exhaustion in lymphoma and leukemia models [194]. P1A tumor antigen-specific TCR-T cells which produced IL7/CCL19, showed noticeably increased anticancer effects and produced long-term memory responses by enhancing the infiltration of dendritic cells and T cells in tumor tissues, including both endogenous T cells and transplanted P1A T cells [195]. GD2-specific CAR T cells were modified to release IL-15 to improve the destruction of lung cancer [196] and glioblastoma [197]. Recently, IL-15-secreting CAR T cells have been investigated to target MDSC in glioblastoma, making CAR T cells more beneficial [198]. These IL-15-secreting CAR T cells were also used in clinical studies for hepatocellular carcinoma treatment (NCT05103631, NCT04377932). In mouse models of small cell lung cancer, IL-18-secreting CAR T cells targeting Delta-like protein 3 induced a persistent response and decreased T cell exhaustion [199]. In a trial at the University of Pennsylvania, IL-18-releasing CAR T cells were investigated for the treatment of CLL, NHL, and ALL (NCT04684563) [200].

### T cell exhaustion in the immunosuppressive TME

The mouse model of chronic lymphocytic choriomeningitis virus infection was the initial experiment to demonstrate T cell exhaustion, in which virus-specific CD8 T cells exposed to ongoing antigen stimulation exhibited diminished effector function and poor proliferative capacity in comparison to functional memory CD8 T cells [201]. Later researches revealed exhausted T (Tex) cells to be a distinct heterogeneous population of immune cells that played a crucial role in the development of cancer, autoimmune diseases, and chronic infections. Effector activities were gradually lost in Tex cells, which also exhibited strong and persistent inhibitory receptor expression, metabolic dysregulation, poor memory recall and homeostatic self-renewal [202]. The effectiveness of ACT in solid tumors was significantly hampered by T cell exhaustion. Decreasing exhaustion to maintain T cell efficiency and durability was a key problem.

Immune checkpoint blockade is an essential method for preventing T cell exhaustion. Currently, drugs and technologies targeting immune checkpoints have been combined with CAR T cells, showing promising results [203–205]. Immune checkpoint blockage temporarily activates Tex cells, but it has no lasting impact on their epigenetic structure. Therefore, a variety of epigenetic markers have been found to contribute to exhaustion when overexpressed or knocked down. This has rapidly become a more general direction to alleviate T cell exhaustion. For instances, knockdown of PR domain zinc finger protein 1 (PRDM1) in CD19-targeting CAR T cells resulted in the production of better-quality T cells, increasing T cell persistence and decreasing T cell exhaustion [206]. Knockout of PRDM1 and NR4A3 improved the anti-tumor response by increasing the generation of long-lived memory cells, counteracting exhaustion in tumor-infiltrating CAR T cells, and enhancing the overall anti-tumor response [207]. The extensive application of CRISPR/Cas9 and bioinformatics-based technologies has provided researchers with the chance to identify the important parameters that could regulate T cell exhaustion. The proliferation of TCR-T cells targeting NY-ESO-1 and M5 CAR T cells targeting mesothelin was increased by the double knockout of Regnase-1 and Roquin-1, which also improved anti-tumor efficacy and T cell lifespan [208]. The constant interaction between CARs and antigens was one cause of CAR T cell exhaustion. With antigens being unregulated, attention has been focused on making CARs controllable [209]. Inducing rest by downregulating CAR proteins with a drug-regulatable system or employing the tyrosine kinase inhibitor dasatinib could improve CAR-T cell effectiveness by preventing or reversing exhaustion [210]. Similar controllable strategies, such as ligand [211] and light [212] controlled systems, have been utilized to regulate CAR expression, but the effect on CAR T cell exhaustion needed to be studied further. Another approach was to select a more appropriate co-stimulatory domain. In comparison to CD28 CAR T cells, 4-1BB CAR T cells proliferated better, showed less exhaustion, had longer effectiveness, and exhibited better in vivo anti-tumor capacity [213].

## Conclusion and perspective

**Fig. 3:**
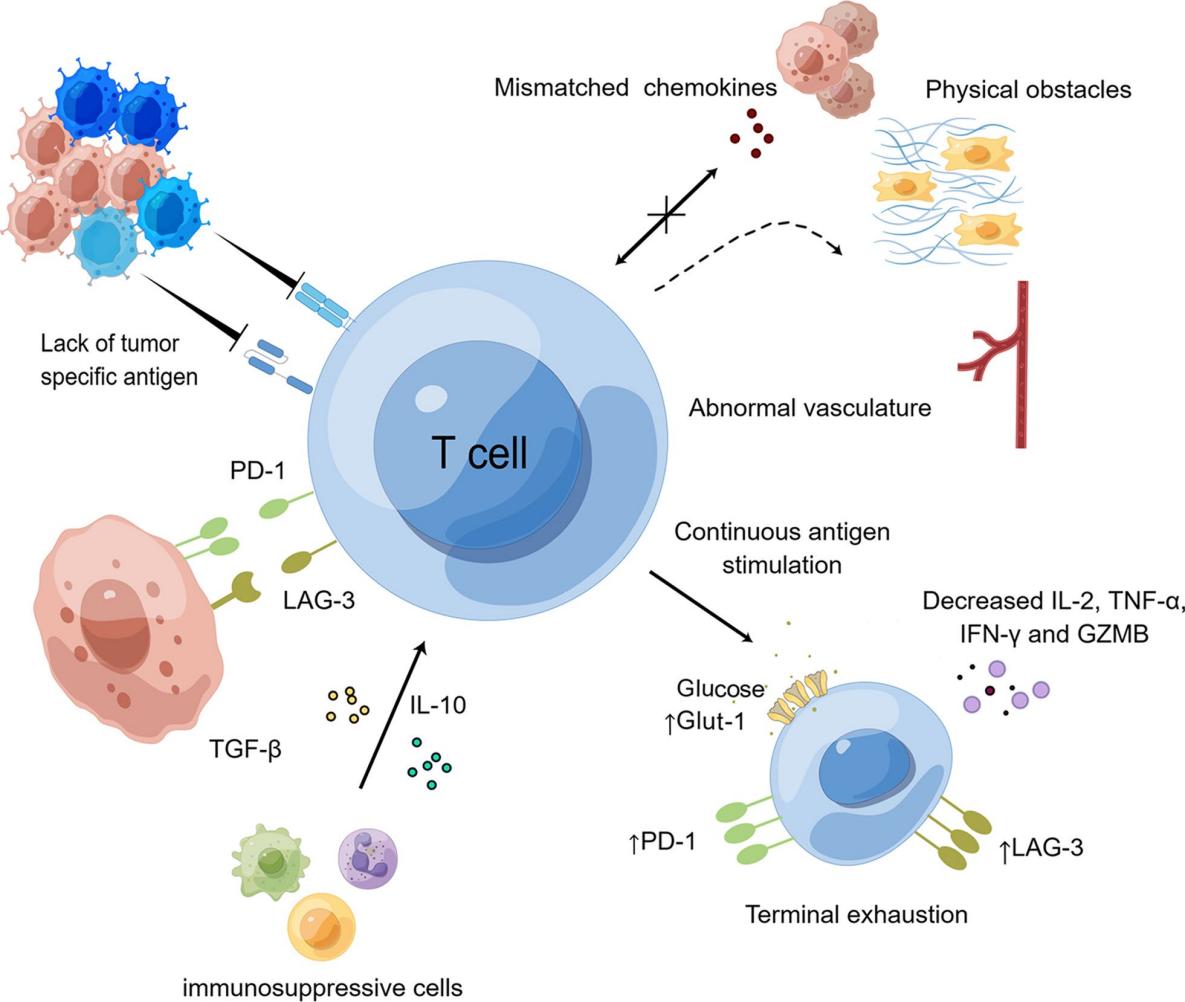
Summary of major barriers of Adoptive cell therapy (ACT) in solid tumors. Some of the most difficult barriers to the development of ACT in solid tumors included tumor heterogeneity, antigen loss, hard trafficking and infiltration, an immunosuppressive tumor microenvironment, and T cell exhaustion. By Figdraw

ACT has shown considerable promise in treating many hematologic cancers, but it has not matched expectations in solid tumors due to a number of constraints, such as antigen loss, poor infiltration in tumors, immunosuppressive TME and so on (Fig. 3). Numerous attempts have been undertaken by scientists to get over these restrictions, and the results have been encouraging. Using bioinformatics and sequencing technology, researchers will be able to identify more specific targets in their work in the future. The infiltration and survivability of effector cells were impacted by the extraordinarily complicated and hazardous internal and external environments of solid tumors. Meanwhile, T cells also became more anergy and exhausted as a result of the aggressive TME and continuous antigen stimulation, which resulted in the failure of ACT. Better understanding the metabolic program and epigenetic states of T cell dysregulation will enhance the efficacy of ACT in treating solid tumors. Despite the remaining obstacles, we believed that ACT would eventually play a significant role in the treatment of solid tumors.

## Data Availability

Not applicable.

## Abbreviations

CTLA-4: Cytotoxic T lymphocyte antigen 4
PD-1: Programmed cell death 1
OS: Overall survival
FDA: The US Food and Drug Administration
ICI: Immune checkpoint inhibitor
ACT: Adoptive cell therapy
TIL: Tumor infiltrating lymphocytes
CAR: Chimeric antigen receptor
TCR: T cell receptor
HLAs: Human leukocyte antigens
TME: Tumor microenvironment
MHC: Major histocompatibility
CTL: Cytotoxic T lymphocyte
GZM: Granzymes
IFN-γ: Interferon-γ
TNF-α: Tumor necrosis factor-α
cDC: Conventional dendritic cell
IL: Interleukin
TIL-Bs: Tumor-infiltrating B lymphocytes
TGF: Tumor growth factor
TLSs: Tertiary lymphoid structures
ORR: Objective response rate
CRR: Complete response rate
NSCLC: Non-small cell lung cancer
ITAM: Immunoreceptor tyrosine-based activation motif
TAAs: Tumor-associated antigens
TSAs: Tumor-specific antigens
CTAs: Cancer testis antigens
MAGE: Melanoma-associated antigen
NY-ESO-1: New York esophageal squamous cell carcinoma-1
SS: Synovial sarcoma
SPEAR: Specific peptide enhanced affinity receptor
NCI: The National Cancer Institute
AEs: Adverse events
TCR-T: T cell receptor-transgenic T
HPV: Human papillomavirus
HBV: Hepatitis B virus
EBV: Epstein-Barr virus
NPC: Nasopharyngeal carcinoma
NHL: Non-Hodgkin lymphomas
NK: Natural killer
PBMC: Peripheral blood mononuclear cells
scFv: Single-chain fragment variable
TRUCKs: T cells redirect for universal cytokine killings
R/R: Relapsed/refractory
B-ALL: B-cell precursor acute lymphoblastic leukemia
LBCL: Large B-cell lymphoma
FL: Follicular lymphoma
DLBCL: Diffuse large B-cell lymphoma
PMBCL: Primary mediastinal large B-cell lymphoma
HGBCL: High-grade B-cell lymphoma
CRS: Cytokine release syndrome
ICANS: Immune effector cell-associated neurotoxicity syndrome
MCL: Mantle cell lymphoma
DOR: Duration of response
MM: Multiple myeloma
BCMA: B-cell maturation antigen
PFS: Progression-free survival
NMPA: National Medicine Products Administration
DIPG: Diffuse intrinsic pontine glioma
GPC2: Glypican 2
ECM: Extracellular matrix
CAFs: Cancer-associated fibroblasts
MMPs: Matrix metalloproteinases
HER2: Human epidermal growth factor receptor 2
FAP: Fibroblast activating protein
EDA: Extra domain A
EDB: Extra domain B
VEGF: Vascular endothelial growth factor
VEGFR: Vascular endothelial growth factor receptor
Treg: Regulatory T cells
TAM: Tumor-associated macrophages
MDSC: Myeloid-derived suppressor cells
LAG-3: Lymphocyte activation gene 3
ICOS: Inducible co-stimulatory factor
rhIL-7-hyFc: Recombinant human IL-7 fused with hybrid Fc
Tex: Exhausted T
PRDM1: PR domain zinc finger protein 1
GD2: Disialoganglioside
